# A Qualitative Analysis of Human–Animal Interactions with Respect to Zoonoses in Nepal

**DOI:** 10.1007/s10393-025-01750-w

**Published:** 2025-08-12

**Authors:** Anna Durrance-Bagale, Hari Basnet, Nanda Bahadur Singh, Steven R. Belmain, James W. Rudge, Natasha Howard

**Affiliations:** 1 Department of Global Health & Development, London School of Hygiene & Tropical Medicine, 15-17 Tavistock Place, London, WC1H 9SH UK; 2 Nepalese Ornithological Union, Kathmandu, Nepal; 3 Central Department of Zoology, Tribhuvan University, Kathmandu, Nepal; 4 Natural Resources Institute, University of Greenwich, Chatham Maritime, Kent, ME4 4TB UK; 5 Nuffield Centre for International Health and Development, Leeds Institute for Health Sciences, University of Leeds, Leeds, LS2 9NL UK; 6 Saw Swee Hock School of Public Health, National University of Singapore and National University Health System, 12 Science Drive 2, Singapore, Singapore

**Keywords:** Human–animal conflict, Nepal, Pest management, Qualitative research, Rodents, Wildlife, Zoonotic disease

## Abstract

Infectious diseases of zoonotic origin are a serious threat to human health and livelihoods globally. Habitat encroachment and deforestation bring humans and animals into contact, increase potential for disease spread, and foster human–animal conflict. Our aim, using thematic analysis, was to qualitatively examine the zoonotic disease landscape in Nepal from public, policymaker, and healthcare practitioner perspectives, and to describe key human–animal interactions. Community participants at six sites were interviewed or took part in focus groups (*n* = 73); 20 healthcare practitioner and policymaker representatives were interviewed. Lack of data complicates understanding of the zoonotic disease landscape in Nepal and limits evidence-informed policymaking. Some participants were aware of the potential significance of Nipah virus in Nepal, but insufficient data precluded planning for potential outbreaks. Drivers of some zoonoses, such as leptospirosis, may be difficult to address as they are related to traditional practices, such as consumption of rodents or barefoot paddy planting. Community participants identified rodents as frequently responsible for human–animal conflict in both rural and urban areas. Most participant photographs included evidence of rodent damage or mitigation against rodents. Habitat encroachment and deforestation have increased wild animal sightings and may increase contact between these and domestic animals, and humans. Although community participants reported no longer killing and eating wild animals, some health/policy participants questioned whether communities adhere to relevant regulations. This underlines the importance of involving communities in culturally appropriate policy development and implementation. To strengthen policymaking around zoonotic disease prevention and human–animal conflict, with the aim of reducing spread of zoonoses, we recommend public engagement between affected communities, healthcare practitioners, and policymakers to agree priorities (e.g. rodent damage and potential mitigation); and further research on effects of anthropogenic environmental changes in conjunction with members of communities most likely to be affected by increased contact with wild animals.

## Introduction

New and re-emerging infectious diseases are serious threats to human health and livelihoods, particularly among those living in resource-constrained countries such as Nepal (Jones et al. 2008). Approximately 75% of these diseases are of zoonotic origin, and many are believed to be sustained in wildlife reservoirs (Lawler et al. 2021; Karesh et al. 2012; Taylor et al. 2001). Data on incidence and prevalence of zoonotic disease in Nepal are lacking, although leptospirosis and rabies are serious long-standing public health issues throughout the Indian subcontinent. Avian influenza was first reported in poultry in Nepal in 2009, with the first human infection in the country reported in 2019 (Acharya et al. 2020a). Nipah virus, an emerging pathogen with a case fatality rate of 70%, is spread by bats, and has been responsible for outbreaks among humans in neighbouring India and Bangladesh following several spillover events (Epstein et al. 2020; Luby et al. 2009). Human encroachment on wild animal habitat and intensive livestock farming are likely drivers of spread of Nipah virus in India, Bangladesh, and Malaysia, as bats’ natural habitat is destroyed and these animals come into contact with livestock that may carry the virus (McKee et al. 2021; Bhowmik et al. 2025). With between 20,000 and 40,000 animal bites reported annually in Nepal—90% from dogs (Pantha et al. 2020)—these animals pose the main rabies threat to humans; however, in 2019 there was a reported death from rabies after a bite from a bat (personal communication, SB Pun, April 2022). Spillover of pathogens such as rabies and Nipah virus at the bat–human interface may be of increasing concern as roosts of Indian Flying Fox (*Pteropus medius*) are present across Nepal (Thapa et al. 2023).

Deforestation and urbanisation, both issues in Nepal, may increase likelihood of contact with commensal rats, which in turn increases potential infection with leptospirosis (Chaudhary et al. 2023; Nava et al. 2017). Leptospirosis is not included in routine surveillance or the early warning and reporting system in Nepal, and incidence is presumed to be under-reported due to a lack of appropriate diagnostic capacities (Costa et al. 2015). This disease likely poses a serious health threat in many areas of Nepal: one study found that 18 of 36 patients diagnosed with enteric fever at a Kathmandu hospital actually had leptospirosis (Murdoch et al. 2004).

Over 80% of Nepalis are engaged in some form of agriculture, with one of the highest livestock-to-human ratios in Asia (5.8 livestock per household). In rural and agricultural areas, the interface between wildlife reservoirs and domesticated livestock may present additional opportunities for pathogen transmission (Johnson et al. 2020; Kelly et al. 2018). An additional factor in zoonotic risk in Nepal is consumption of bushmeat. Rats and bats are hunted and eaten in some communities, especially during festivals (Mickleburgh et al. 2009), although it is not known how widespread these behaviours are within these groups.

Human–animal conflicts caused by habitat encroachment, particularly in forest areas, and competition for food and other resources (Gaire and Acharya 2023)—exemplified by crop and property damage, and predation on livestock (Rawal et al. 2024; Karki et al. 2022; Baral et al. 2021)—are a significant issue in Nepal. Similarly to the situation around zoonotic disease, data on temporal and spatial patterns of human–animal conflict are lacking (Acharya et al. 2016). One study found that, over a 5-year period, 27% of livestock owned by people surveyed in two districts was killed by wild animals, equating to a loss of over US$140 per household (Baral et al. 2021). Crops were destroyed by rhesus monkeys (*Macaca mulatta*; responsible for 74% of reported damage) and field mice (*Mus booduga*; responsible for 12%). In a country where 2024 per capita gross domestic product was US$1,400, this loss is significant (International Monetary Fund. Nepal GDP per capita.^2024^ [Available from:https://www.imf.org/external/datamapper/NGDPDPC@WEO/NPLzoom=NPLhighlight=NPL^2024^).

In terms of domestic interactions, Nepalis report frequent contact with commensal rodents such as rats and mice, and more infrequent contact with bats, suggesting that this is a serious issue that needs to be addressed, both in terms of disease prevention and protection from crop, household, or physical damage (Durrance-Bagale 2024). Community dogs are also ubiquitous throughout Nepal, with the public often aware of the potential role of these animals in transmission of rabies (Durrance-Bagale et al. 2025).

We aimed to examine the zoonotic disease landscape in Nepal from public, policymaker, and healthcare practitioner perspectives, and to describe key human–animal interactions that may impact spread of zoonoses in different regions of the country.

## Methods

### Study Design

This qualitative multimethod study incorporated interviews, focus group discussions (FGDs), and photovoice with members of the public, and interviews with health-workers, veterinarians, and policymakers. Our research questions were: ‘what experiences have participants had with animals (domestic and wild)?’ and ‘how do health professionals and policymakers perceive zoonotic disease in Nepal?’.

### Study Sites

**Figure 1:**
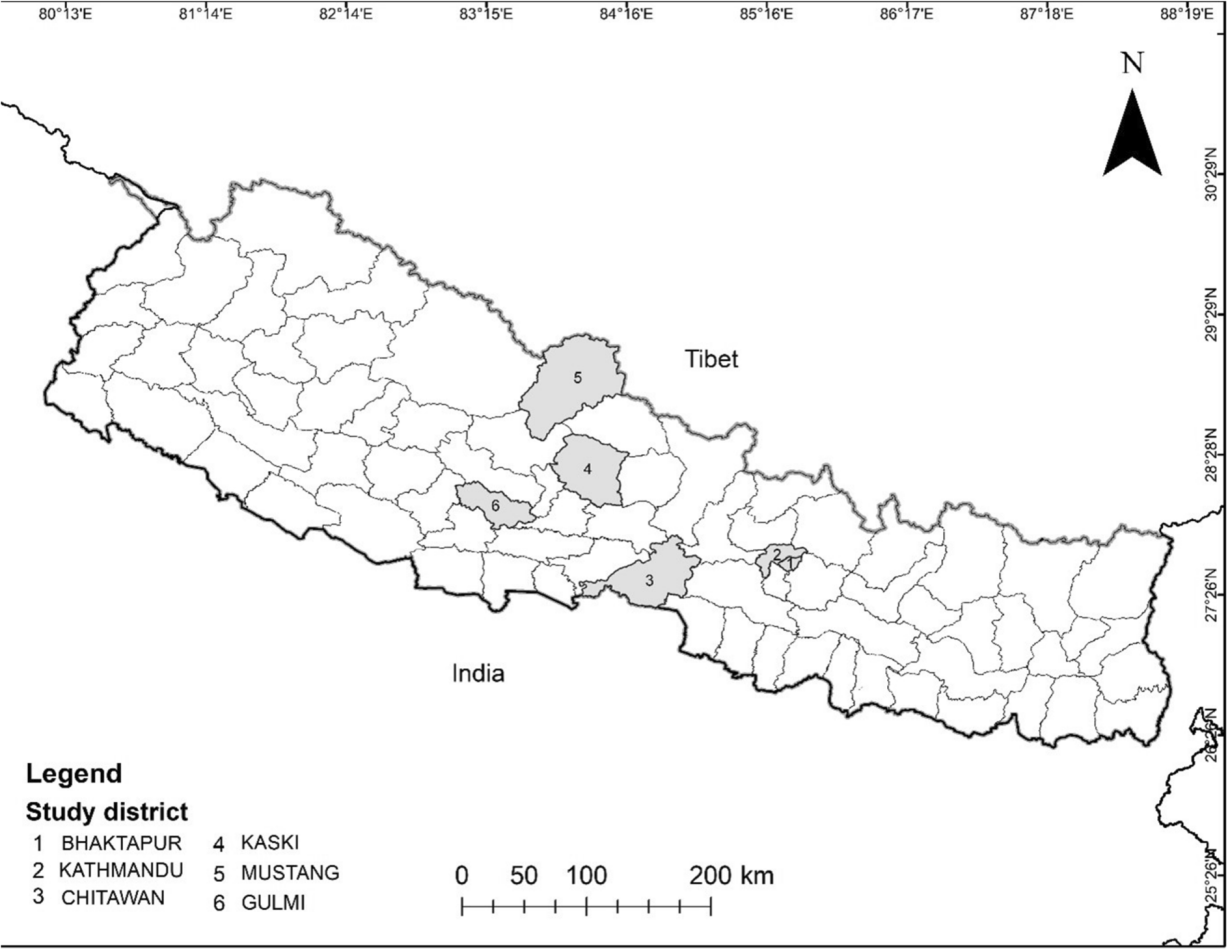
Map of Nepali districts included in study.

Through discussion with Nepali colleagues, we purposively selected 6 sites where participants were likely to provide information-rich interviews, including 2 in the Kathmandu valley (1 of which was an informal settlement; Fig. 1).

### Participant Sampling and Recruitment

At each site, we used typical sampling and snowballing to select adult participants (6–8 individual interviews and 6–10 individuals per FGD) likely to generate rich data to help answer our research questions. To identify the first participant in each community, ADB and HB contacted a healthcare worker or prominent community member (e.g. women’s group member, teacher), and asked them to suggest names. Each participant was then asked to suggest other potential participants.

We generated a seed list of Nepali human or animal healthcare professionals and national or regional policymakers, and asked Nepali colleagues to nominate potential participants from relevant organisations. Three people on the seed list did not respond and 2 refused due to time constraints. Participants were also asked to nominate others who could make relevant contributions.

### Consent Procedures

ADB and HB explained study details to potential participants and asked them to sign a consent form or give verbal consent confirming they had read and understood the information sheet. Consent given by participants with low literacy was witnessed by someone selected by them and unrelated to the study team, after thorough explanation of the information sheet.

### Data Collection

ADB and HB collected data between April and July 2022. Both were trained in qualitative research methods and were experienced at conducting this type of research. All participants were interviewed in a place of their choice (e.g. home, workplace, local café) to improve confidentiality and privacy. Recordings and photographs were given an alphanumeric code. Completed consent forms were scanned and shredded. Electronic data were password-protected and only accessible to the study team.

#### Interviews and FGDs

The community participant topic guide examined human–animal contact, biosecurity and food hygiene, environmental changes, health issues, and disease awareness. The policymaker and practitioner topic guide examined relevant experience, views on community awareness, and governmental policy on zoonotic and infectious diseases. Topic guides were informed by findings from a literature review (Durrance-Bagale et al. 2021) and team discussion of the context. Participants were encouraged to raise additional topics. Interviews took approximately 30 min and were conducted in English (by ADB) or Nepali (by ADB and HB) depending on participant preference. ADB conducted 7 policymaker and healthcare professional interviews remotely using Zoom software (Zoom Video Communications Inc., San Jose), audio-recorded with automatic transcription enabled. HB and ADB conducted FGDs in Nepali, taking around 45–60 min. One FGD was conducted in both Nepali and Newari at participants’ request, as all were fluent in both languages.

#### Photovoice

We used photovoice as a collaborative approach to data collection (Glaw et al. 2017) to enhance understanding of people’s experiences, beliefs, behaviours, and priorities (Catalani and Minkler 2010). Participants take images and are then asked to explain the relevance of these images for them. We determined that photovoice could be useful in the marginalised communities we included, members of which were less familiar with traditional methods of enquiry such as interviewing (Mukumbang and van Wyk 2020). After interview, participants who appeared enthusiastic about the topic were given a simple digital camera to take photographs illustrating their feelings or perceptions of zoonotic disease risk in their community. They were asked to describe why they had taken these images, and these explanations are included with the images below.

ADB transcribed English language interviews. Recordings in Nepali were transcribed into English by a Nepali healthcare professional familiar with public health and infectious disease, with training in transcription, and fluent in both languages. Two transcripts were reviewed and back-translated by another native Nepali speaker to ensure text reflected recordings. ADB cross-checked names used for animal diseases with a Nepali veterinarian fluent in English to ensure they represented the correct disease as far as possible, and to check for any nuance related to these diseases that might not have been obvious to the transcriber.

### Analysis

We imported all transcripts into NVivo software (QSR International Pty Ltd, Version 12, 2018) for data management. ADB assigned all photographs an identifying label and textual description, which was imported into NVivo with accompanying explanatory text from the participant.

ADB reviewed and coded transcripts, photograph summaries, and observation notes in NVivo, using deductive and inductive reflexive thematic analysis to generate themes and sub-themes from the data (Braun and Clarke 2006, 2021). Two of the co-authors independently reviewed the coding. The six analysis steps were: (i) data familiarisation; (ii) generating initial codes; (iii) generating themes; (iv) reviewing potential themes; (v) defining/naming themes; and (vi) synthesising findings in discussion with co-authors (Braun and Clarke 2006, 2021).

### Ethics

The Nepal Health Research Council (ref: 2193) and London School of Hygiene & Tropical Medicine Observational Research Ethics Committee (ref: 26,507) provided ethics approval. Our study brought together authors from the UK and Nepal, all of whom were engaged from the beginning with study design and research methodology to ensure different perspectives and skillsets were considered.

## Findings

### Participant characteristics

As shown in Table 1, 39 people (21 M/18F) from 6 settlements participated in individual interviews while 34 (14 M/20F) participated in 5 FGDs. Nine interview participants and 1 FGD participant shared photographs across the 6 sites. Twenty Nepali healthcare professionals and policymakers also provided interviews (Table 2): 14 representing human health (13 M/1F) and 6 representing animal health (3 M/3F).

**Table 1:** Community participant characteristics

Identifier	Gender	Estimated age	Language
Bhaktapur1	Female	45–50	Nepali
Bhaktapur2	Female	50–55	Nepali
Bhaktapur3	Male	35–40	Nepali
Bhaktapur4	Female	30–35	English
Chitwan1	Male	45–50	Nepali
Chitwan2	Male	70–75	Nepali
Chitwan3	Female	45–50	Nepali
Chitwan4	Male	40–45	English
Chitwan5	Male	35–40	Nepali
Chitwan6	Male	60–65	Nepali
Gulmi1	Female	65–70	Nepali
Gulmi2	Female	35–40	Nepali
Gulmi3	Female	55–60	Nepali
Gulmi4	Female	45–50	Nepali
Gulmi5	Male	60–65	Nepali
Gulmi6	Female	55–60	Nepali
Kathmandu1	Male	25–30	Nepali
Kathmandu2	Male	45–50	Nepali/English
Kathmandu3	Female	35–40	Nepali
Kathmandu4	Female	35–40	Nepali
Kathmandu5	Male	20–25	English
Mustang1	Female	45–50	Nepali
Mustang2	Female	40–45	Nepali
Mustang3	Male	20–25	Nepali
Mustang4	Male	40–45	Nepali
Mustang5	Female	35–40	Nepali
Mustang6	Male	40–45	Nepali
Mustang7	Male	45–50	Nepali
Pokhara1	Male	45–50	Nepali
Pokhara2	Male	20–25	Nepali
Pokhara3	Male	30–35	Nepali
Pokhara4	Female	50–55	Nepali
Pokhara5	Male	45–50	Nepali
Pokhara6	Female	50–55	Nepali
Pokhara7	Male	55–60	Nepali
Pokhara8	Male	55–60	Nepali
Pokhara9	Female	50–55	Nepali
Pokhara10	Female	35–40	Nepali
Pokhara11	Male	55–60	Nepali
Bhaktapur FGD	5 male, 4 female	20–70	Nepali/Newari
Chitwan FGD	1 male, 3 female	20–70	Nepali
Gulmi FGD	0 male, 9 female	20–70	Nepali
Mustang FGD	5 male, 1 female	20–70	Nepali
Pokhara FGD	3 male, 3 female	20–70	Nepali

**Table 2:** Healthcare professional participant characteristics

Identifier	Type	Gender	Interview
Health1	Infectious disease specialist	Male	In-person
Health2	Clinician/NGO	Male	In-person
Health3	Public health specialist	Male	In-person
Health4	Consultant for health NGOs/iNGOs	Male	In-person
Health5	Government (central)/NGO	Female	In-person
Health6	Infectious disease specialist	Male	In-person
Health7	Government (central)/clinician	Male	In-person
Health8	Consultant for health NGOs/iNGOs	Male	In-person
Health9	Government (central)/infectious disease specialist	Male	In-person
Health10	Consultant for health NGOs/iNGOs	Male	Remote
Health11	Infectious disease specialist/academic	Male	Remote
Health12	Public health specialist	Male	Remote
Health13	Consultant for health NGOs/iNGOs	Male	Remote
Health14	Government (regional)/public health specialist	Male	Remote
Livestock1	Government (central)/veterinarian	Male	In-person
Livestock2	Government (central)/veterinarian	Male	In-person
Livestock3	Government (regional)/veterinarian	Female	In-person
Livestock4	Government (regional)/veterinarian	Male	In-person
Livestock5	Government (regional)/veterinarian	Female	Remote
Livestock6	Government (central)/veterinarian	Female	Remote

*iNGO* international non-governmental organisation, *NGO* non-governmental organisation

### Thematic analysis

We generated 2 overarching themes: (i) zoonotic disease landscape, and (ii) interactions with domesticated, wild, and commensal animals. Relevant quotes are presented under the sub-headings below, grouped by participant type (community or practitioner/policymaker).

### Zoonotic disease landscape

Sub-themes included lack of data on the zoonotic disease landscape in Nepal; Nipah virus disease; rabies; leptospirosis; and avian influenza. Nipah virus disease and rabies were most frequently discussed by healthcare practitioners and policymakers, with some participants mentioning leptospirosis and avian influenza.

#### Lack of data on zoonotic disease

Clarifying the zoonotic disease landscape in Nepal is complicated by a lack of data, although infectious diseases are recognised by Nepali health professionals to be a huge burden on an under-funded, under-resourced health system:

‘The major infectious disease in Nepal, they are related to tuberculosis, HIV, and also ARI, acute respiratory infections[…]Nepal is also prone to zoonotic disease, so many times the health system is experiencing those shocks related to outbreaks, such as influenza outbreak, and even scrub typhus[…]COVID is also a zoonotic disease. So it's troubling the health system right now.’ [Health3]

One senior regional health official used humour to try and underline the poignant reality of the situation:

‘[*Redacted region*] itself is an infectious disease community lab. I always say to the researchers that you can find every infectious disease here[…]if you can’t find any infectious disease somewhere you can come to [*redacted region*] and you will find everything [*laughing*].’ [Health14]

A further complication is that non-communicable diseases (NCDs) attract more funding and publicity and are often prioritised over other diseases. NCDs have more measurable targets, allowing politicians to demonstrate progress to funders and international partners. The lack of zoonoses-focussed resources leads to a lack of data that could inform research and programming implementation:

‘Most of the disease comes from the NCDs, mental health issue, and sometimes people forget the infectious disease. But they will never die out. That means we need to take the lesson from COVID-19. I think there is a big problem of the infectious disease and we need to sustain our achievement we have made in the past. And that means they [*government*] should not cut off the resources and we need to still increase the infectious disease rates and control.’ [Health7]

#### Nipah virus disease

The potential for Nipah virus to enter Nepal was judged low by one health professional who suggested that relevant cultural practices are not present in Nepal, but admitted that risk is always there:

‘We have bats but the culture of eating these palms, I don't think that is the practice[...]In Terai region also there are bats, so there is risk. There is always the risk.’ [Health6]

However, one prominent zoonotic disease expert disagreed, stating that as Nipah virus is present in neighbouring countries there is potential for the virus to cross the border, especially as the bats believed to be the virus vector live in the centre of Kathmandu:

‘I’m extremely worried about it [*Nipah virus*] because we’re sharing a border with India and they detect it frequently, and in Bangladesh[…]unfortunately, that kind of bat is actually in Nepal.’ [Health1]

Another healthcare practitioner suggested that Nipah virus could already be present in the country, in humans, or in animal reservoirs (e.g. bats), but that data are not available to refute or confirm the hypothesis:

‘Nipah is of no priority because people say we don't have cases, but we have bats[…]infected people may travel, because so many Nepalese are going to Malaysia, Bangladesh, India and coming back, right? We need a study, then only we can say 'yes, we don't have'.’ [Health7]

Cultural practices that may encourage spread of Nipah were discussed by one central government participant:

‘In Nepal, there are some communities that hunt and then eat bats like Chepangs[…]definitely through that unnatural system also there is some possibility, but if the disease is established in India, then definitely through the people’s movement, it can come anytime into Nepal.’ [Livestock2]

This was underlined by a second participant working for the government, who related transmission to the cultural practice of drinking palm sap, which is often reported as a driver of outbreaks in Bangladesh.

Another central government participant discussed the increasing number of pigs being farmed in Nepal, and, as pigs are an amplifying host for the virus, suggested that this could encourage the spread of Nipah, particularly as the farming area is contiguous with India:

‘The interesting thing is the pig industry is growing where Nepal meets India in the eastern side[…]we are kind of inviting the Nipah virus. We have a lot of pigs in this side, and it's where we need to be very alert, we need to be in high alert.’ [Livestock1]

#### Rabies

Rabies was acknowledged as a serious issue in Nepal, with prophylactic vaccination administered free-of-cost. Healthcare participants stated that most people know at least something about how to prevent the disease, or what to do if bitten. However, they discussed problems around trying to control infection, including vaccination hesitancy, and lack of recognition from human healthcare professionals:

‘Rabies has a big burden. Even they [*human healthcare professionals*] don't like to diagnose. It's difficult for them to diagnose. But I have a lot of experience of rabies in animals. Mostly dog, pig, cattle. Even horse.’ [Livestock4]

The animal health sector, both government departments and non-governmental organisations, was involved in promoting information about rabies to the public:

‘We celebrate this World Rabies Day, every year. So it helps in a way. And it's not just the government that's working in it. We have a lot of animal welfare organisations, which work on rabies, you can find a number of them, and they have been contributing to it.’ [Livestock1]

Two infectious disease specialists framed the lack of successful control of rabies as a political issue, suggesting that, again, politicians do not give enough attention to the disease but instead are concentrating on NCDs, and that rabies needs to be notifiable to allow effective control:

‘If somebody is bitten by the dog, they ignore. ‘It is a very small wound, why should I have to go [*to the hospital*]?’ Still we have to do hard work to convince them [*politicians*] that zoonotic diseases are one of the very serious public health problems in Nepal. So we are still failing, and the concerned persons are really not giving attention to these diseases. Instead they are focusing on heart disease or kidney disease or whatever as their priority.’ [Health1]

#### Leptospirosis

Both human and animal health practitioners discussed leptospirosis in Nepal, with one veterinarian suggesting that the cultural practice of consuming rodents during festivals complicates the issue of changing behaviour around the disease:

[‘*Do people know about leptospirosis?’*] ‘By name? Definitely they don't. But maybe they might know from the local name[…]since it's like after every monsoon, this type of problem, there might be people have some knowledge on that, but definitely as *Leptospira* they don't have that.’ [Livestock2]

‘Rats have many diseases. We have reports of leptospirosis in animals and in humans. So that might be a burden, but people they have a culture, they have a tradition that after they harvest the paddy, they keep on digging and digging to get the rats. They make the rat like a barbeque.’ [Livestock4]

#### Avian influenza

Avian influenza has the potential to negatively affect many backyard farmers in Nepal who keep poultry to supplement their diet. This is being addressed through revised regulations and policy, although there is debate about the significance of the disease in the country, with participants from both central and regional government discussing the familiar issue of lack of data:

‘Nepal faced the first outbreak of avian influenza in 2009. And that was kind of a turning point for us. And before we had the outbreak, we had this preparedness plan for avian influenza and contingency plan kind of thing[…]we recently had this bird flu control regulation approved by the Government of Nepal[…]based on that regulation, we have been conducting control activities for avian influenza.’ [Livestock1]

### Interactions with Domesticated, Wild, and Commensal Animals

Sub-themes around human–animal interactions included pest management; consumption of wild animals; and contact with wild animals.

#### Pest Management

**Figure 2:**
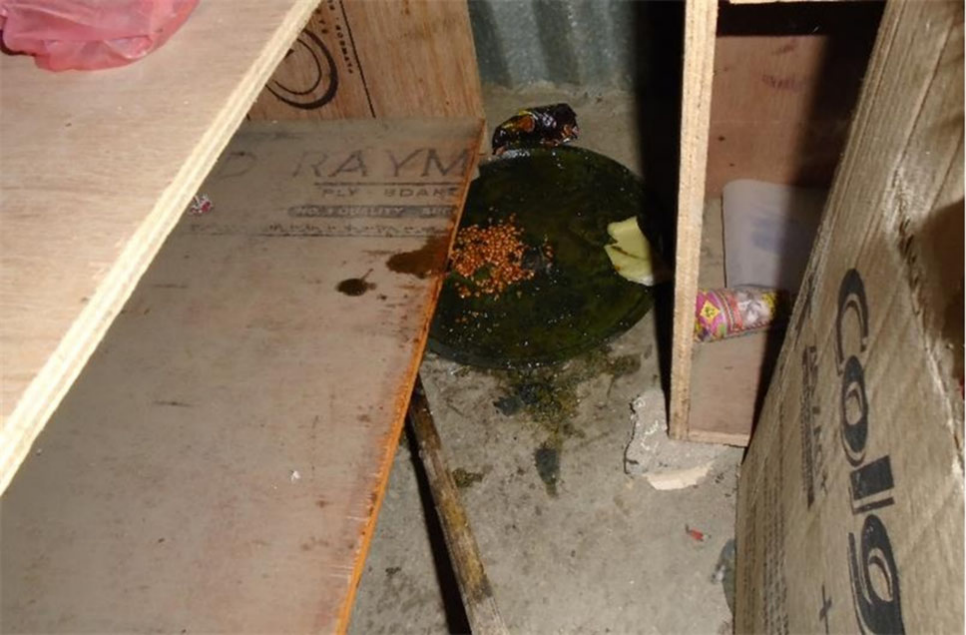
Home-made glue trap (Kathmandu3).

Community participants considered rodents to be the main nuisance animal in their environment, destroying property (clothes, furniture, grain) and crops in fields. They discussed methods of managing rodents in and around their community, including use of traps and poison, and efficient disposal of their corpses. Photographs showed home-made rat traps, household damage, faeces in food storage, and disposal of dead rodents. A shopkeeper in the informal settlement in Kathmandu discussed the damage rodents caused to her stock. She tried conventional traps to prevent this damage but the rats were so large they were able to drag themselves and the trap out of the shop. She photographed her home-made rodent trap, made from a piece of round, flat metal covered in lentils and glue (Fig. 2). Once the rats were stuck on the metal, she killed and threw them in the river running beside the settlement.

**Figure 3:**
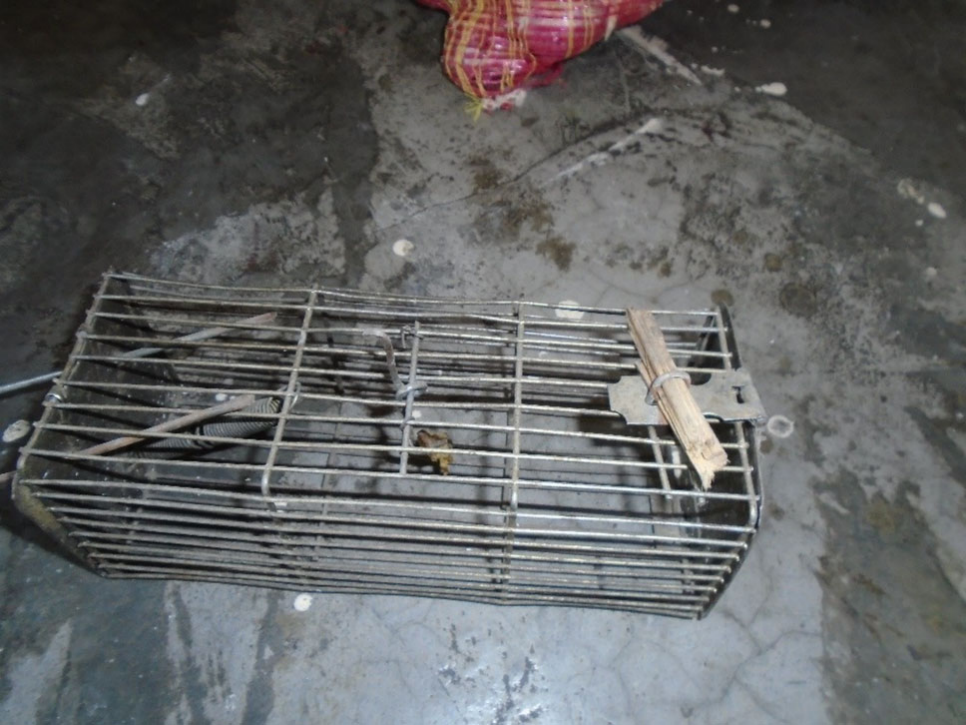
Rodent trap (Mustang1).

A participant in Mustang took a photograph of a more conventional rat trap, commenting that: ‘The rats are so strong as it collects a lot of grains.’ [Mustang1; Fig. 3].

**Figure 4:**
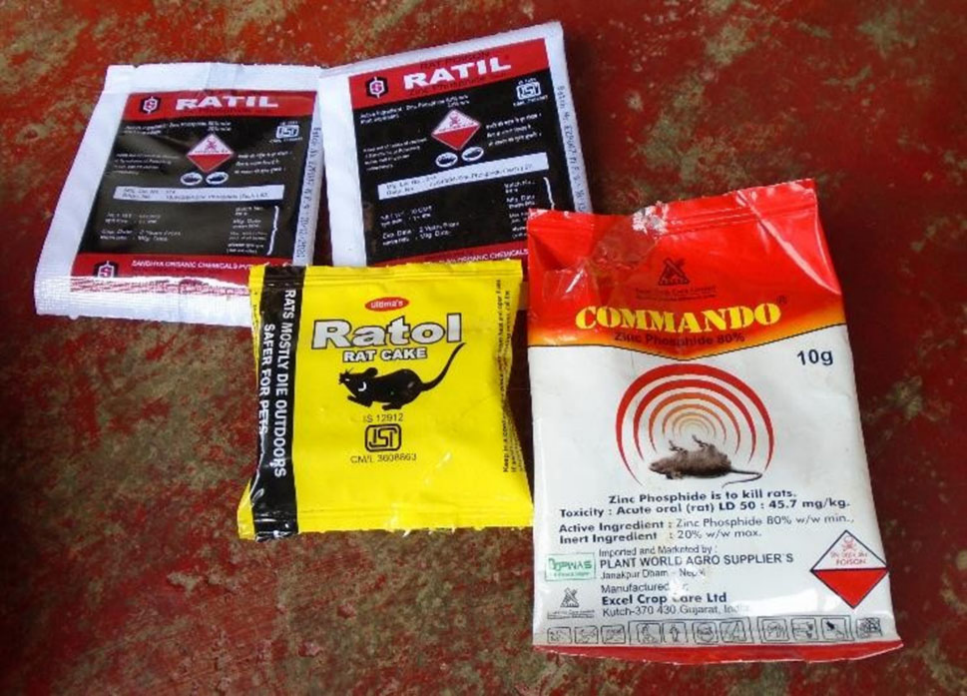
Selection of rodent poisons (Pokhara11).

Other measures against pest animals included the use of poison, as demonstrated by one photovoice participant who took a photograph of the substances used in his house to control rodents (Fig. 4). When asked to explain why he killed rodents, his response related to disease, specifically rabies.

A participant who ran a farm mentioned the use of traps and poison, and how controlling rodents was a topic farmers discussed together:

‘In the field we find wheat mixed with poison that we keep near the hole that the rat eats and dies. In the house we use traps and also, we mix meat with wheat balls mixed with poison to kill it. We also discuss with other workers to control rats.’ [Pokhara1]

All participants who mentioned disposal of dead rodents emphasised that this was done as far away as possible, sometimes by village sweepers, or that corpses were sometimes pragmatically added to the gobar gas producer (biogas produced from cow or buffalo dung), converting them to a useful source of energy:

‘As I don’t use any poison I put the dead mouse in the gobar maker. We do not put the dead mouse that we find around the house in the gobar gas maker and usually bury them outside because they can be poisoned, which can affect the bacteria in the gobar gas.’ [Pokhara7]

Although widespread, the disadvantages of using poison were recognised, including the smell of decomposing rats and danger of inadvertently poisoning livestock, with one participant suggesting that their community stopped eating rats in case they had been poisoned.

**Figure 5:**
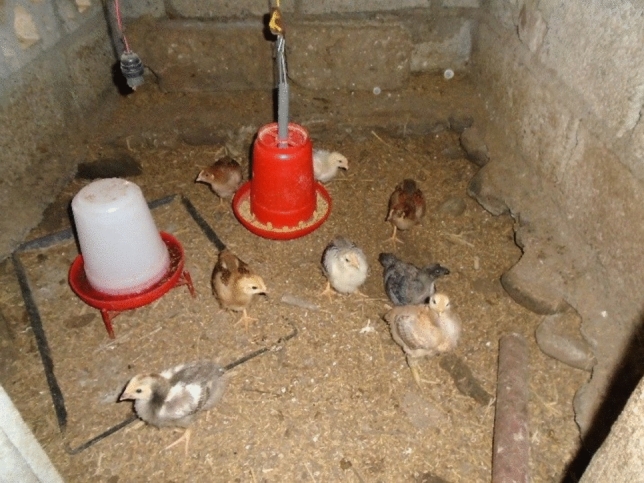
Chickens at risk of rodent bites (Pokhara8).

Rodents may attack livestock in people’s homes, as photographed by one participant, who told us his chickens have been bitten by rodents, which he thought might be attracted by the easily accessible food (Fig. 5).

**Figure 6:**
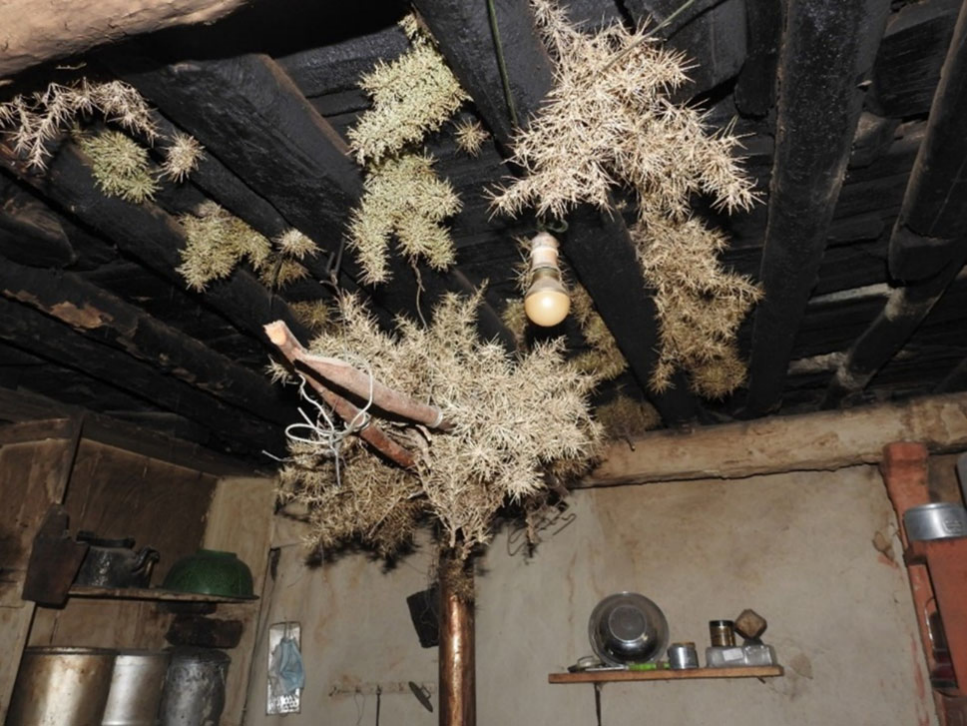
Thorns used to prevent rodents eating yak meat (photograph by Hari Basnet).

Some mitigatory activities were specific to certain regions or cultures, e.g. participants in the Mustang FGD talked about using a local thorny plant (Fig. 6) to protect their meat from rodent damage:

‘As they not only get into food but also cut the clothes it is important to control it[…]We have a culture of cutting yak and while we are storing the yak meat, we use these thorns that prevent the rats from getting to the meat. These thorns are very sharp and thin.’ [Mustang FGD]

#### Consumption of Wild Animals

Many participants discussed consumption of wild animal meat (e.g. monkey, deer, bat), often as typical of other communities, although some mentioned that this practice is generally decreasing due to conservation measures:

‘Not here in Kathmandu or around here but when I visited the places outside the valley, I knew people eating wild animals indeed. We stayed with the family or the community where they were consuming bat. It was in Chitwan. The Chepang community.’ [Bhaktapur4]

‘I had wild chicken [*Khalij pheasant*] before. Nowadays we do not find it here but there are wild chicken meats that people sell in the village. People also consumed deer but now hunting of deer is restricted here so we have not consumed these meats.’ [Pokhara3]

Some healthcare practitioners discussed their understanding of wildlife poaching, and how this relates to cultural practices and beliefs, such as traditional healing:

‘It is a big issue in the buffer zones, so mostly the people they just poach wild boars and deer. Sometimes they use an electric current or sometimes they use blasting. Still they consume. It’s common in the buffer zone, not everywhere. And have you heard about drinking of blood of yak? In Mustang, they have a festival. They have a tradition that on that day, they don’t slaughter the yak, they cut the neck and drink the blood, and they believe that it makes them healthier.’ [Livestock4]

#### Contact with Wild Animals

The potential for infectious agents to transmit from wild animals (e.g. monkeys) to either domestic livestock or humans as a result of habitat encroachment and human population increase was discussed in detail by some practitioners:

‘I think Nepal is quite vulnerable to zoonotics because we have a lot of factors that are quite favourable for the emergence or re-emergence of zoonotic disease[…]because the population is increasing, and we are encroaching on a lot of forest areas[…]if you go to Chitwan National Park, you do not feel like it's a national park anymore. You feel like it's an urban centre, this is a city and then just a few forest areas, that's a clear example of how human lives are encroaching on natural habitats of wild animals[…] We have a very open and porous border with India. So any sick animal can cross the border. Given all these factors, I'd say we’re very vulnerable to zoonotic disease.’ [Health5]

The close proximity of human habitation to forested areas was a concern for community participants too, who reported seeing more wild animals in recent years:

‘The density of forest has increased and the number of wild animals also increased. Monkey have been giving us problems. Red deer also come out. We also see wild boar that we killed and eat[…]we started seeing red deer about 3 years ago, also porcupine have been seen around.’ [Gulmi2]

‘Hunting of the animals is prohibited and the jungle areas have expanded. Animals that were almost endangered have also started increasing along with the expansion of the forest. Khalij pheasant disappeared from this region but now they are coming back, even Ghoral or Himalayan blue sheep. They are seen quite close to our home.’ [Mustang3]

Bats are present in many communities in Nepal, and potentially an issue as neighbouring countries Bangladesh and India experience frequent Nipah virus outbreaks. Public participants mentioned seeing bats in their communities, and described how they are used in traditional medicine:

‘They [*bats*] come around here that are blackish brown in colour[…]These bats do not stay inside the house and only come during night-time. Our ancestors used to kill the bat and make a necklace out of it called *Jantar* that is believed to treat body aches.’ [Mustang FGD]

‘We also see bats around at night in the cow sheds. It sometimes bites the horns and tails of calves as they have soft and new horns that are not hard.’ [Pokhara11]

## Discussion

Infectious diseases of zoonotic origin are a serious threat to health and livelihoods globally. Anthropogenic changes, such as habitat encroachment, deforestation, and urbanisation, may bring humans and animals into closer proximity and increase potential for disease spread, with negative consequences for humans and animals alike. To our knowledge, this is the first study to examine the zoonotic risk landscape in Nepal with reference to human–animal interactions, from the perspective of policymakers, healthcare practitioners, and public participants in different locations.

### Key Findings

Most healthcare practitioners and policymakers discussed how the lack of sufficient data and available resources complicated understanding of the zoonotic disease landscape in Nepal and did not allow effective policymaking, even with the political will to change the situation. Some participants were aware of the potential significance of Nipah virus spread into Nepal, but lack of data did not allow planning for potential outbreaks. Some zoonoses, such as leptospirosis, may be difficult to address as risks of exposure are related to cultural beliefs and practices, such as consumption of rodents and barefoot paddy planting, which are both significant facets of festivals in different communities in the country (Durrance-Bagale 2024).

Human–rodent conflict (destruction of crops, clothing and property, concerns around infection, and disease spread by rats) was mentioned frequently during community interviews, and most photographs featured either rat-mediated damage or attempts to mitigate such damage (e.g. home-made traps, use of different poisons) in both urban and rural areas, and among both male and female participants, regardless of age. This type of conflict is not specific to Nepal (Scobie et al. 2023), and identifying potential solutions is complicated by the need to involve representatives of different sectors, as well as community members who are well-placed to discuss the effects such incursion has on their lives.

In terms of other interactions with animals, some participants suggested that sightings of wild animals such as deer, monkey, and wild boar had increased in recent years. Although village participants reported no longer killing and eating wild animals, some practitioner/policymaker participants questioned whether everyone is following the regulations.

### Policy Implications

As discussed by many participants, lack of data on the zoonotic disease landscape in Nepal is related to a lack of sufficient financial resources. Prioritisation of NCDs, which are potentially more attractive to funders as they have measurable targets and can more easily demonstrate progress, means that a concerted effort needs to be made to give more focus to zoonoses. A 2025 review of Nepal’s health system found limited systemic capacity, lack of preparedness to focus on changing health priorities, and an insufficient investment in the health system overall: Nepal currently allocates only 2% of gross domestic product to healthcare, which leads to huge competition for available resources (Khatri et al. 2025).

A key policy implication of this research is the need to effectively address impacts of rodents in Nepal. While rodents are important vectors of disease (e.g. leptospirosis, rat-bite fever, scrub typhus), more research on the drivers and burden of these diseases in the country is needed (Acharya et al. 2020b). The chief of the infectious disease hospital in Kathmandu, Dr Sher Bahadur Pun, has been vocal about educating people on the potential effects of rat bites, while recognising that the regularity of these bites may desensitise people to the risks (Pun 2024). This underlines the importance of promoting awareness of potential threats from rodents within communities. In addition, little is known about the socio-economic consequences of zoonoses that may be transmitted from rodents to humans in Asia (Singleton et al. 2021)—studies investigating this facet of rodent–human conflict should be instituted as a priority, to inform policy and mitigate impacts on livelihoods, particularly among the poorest, rural populations who are likely to be most vulnerable.

One relevant example of how contact between wild animals and livestock may increase likelihood of disease transmission between species is Nipah virus. As discussed by one animal healthcare participant, increasing numbers of pigs are being farmed in areas of Nepal with bats (believed to be the virus reservoir) also present. A systematic review of agriculture, environmental change, and zoonoses found that bat-to-pig and pig-to-human transmission has probably sporadically occurred in Malaysia for many years but that more intensive pig farming has recently led to more human infections (Jones et al. 2013). A similar review, focussed on highly pathogenic avian influenza, as discussed by some healthcare participants, also highlighted the anthropogenic nature of increasing risk of zoonotic disease spread from wildlife to livestock to humans (Pfeiffer et al. 2013). This research clearly demonstrates the potential for changes in the environment or in farming practices to affect risk of spillover of disease from animals to humans. Implementing projects to help smallholders mitigate the risk of infection transmitting from wildlife to their livestock, and then to farmers themselves, could help those living in rural areas protect themselves from zoonotic disease.

It is vital that any proposed policies recognise the importance of cultural, religious, and other contextual factors that might influence potential mitigatory practices. One example is the taboo around the use of indigenous predatory animals (snakes and owls) among some communities in Madagascar, where associations of these animals with witchcraft decrease the acceptability of such strategies for rodent control (Scobie et al. 2023). In our study, when participants discussed methods of preventing rodent damage, this was usually framed as necessary to reduce financial loss, rather than benefit human health. People understandably prioritised having enough to eat over becoming ill due to contact with rodents or other animals. Such social norms must be considered when planning interventions or policies.

One way of increasing the potential efficacy of policies or interventions such as awareness campaigns is to involve affected communities in identifying priorities (Durrance-Bagale et al. 2025; Farr et al. 2021; Tugendhaft et al. 2021). Policies that actively engage the public from the planning stage onward may be more effective, as they are more likely to be culturally and contextually sensitive and more realistic, in terms of available resources (Durrance-Bagale et al. 2025; Farr et al. 2021; Tugendhaft et al. 2021). People may already have solutions to issues perceived as problems. For example, in our study, the Mustang community discussed use of thorns to protect yak meat from invading rodents. Communities must be involved in culturally appropriate policy development and implementation, to identify potential drivers of human–animal contact, and reduce concomitant risk of disease spread.

### Limitations and Strengths

Several limitations should be considered. First, we could only include 6 sites: perspectives in other areas of Nepal may differ. However, we did find many practices and beliefs congruent across different geographical areas. Second, some participants may have felt uncomfortable discussing issues they perceived to be sensitive or that potentially could cause them problems (e.g. killing and consuming wild animals in conservation areas). This was especially true as one of the interviewers was a different ethnicity to them, and the process of interviewing was new to most participants. However, we spent time building trust and helping participants feel relaxed with us, and stopped discussing topics if they demonstrated any signs of discomfort. Third, most community interviews involved simultaneous translation. However, all recordings were transcribed verbatim and included both original Nepali vocabulary (translated into English) and simultaneously translated English, to reduce potential loss of meaning. Fourth, not everyone participated in photovoice, which may limit how representative the photographs and accompanying explanations are of the views of the wider population. However, we feel we have demonstrated the utility and acceptability of this method in this type of research. We believe this study contributes to the limited body of evidence on the zoonotic disease landscape and human–animal contact in Nepal.

## Conclusion

Lack of sufficient financial resources, which impacts surveillance and availability of relevant data, impacts of deforestation and urbanisation, which bring people and domestic animals into closer contact with wild animals, and clarification of the causes and effects of animal–human contact are key issues that must be addressed to protect the health of both humans and animals in Nepal. To strengthen policymaking around zoonotic disease prevention and human–animal interactions, with the aim of reducing spread of zoonoses, we recommend public engagement between affected communities, healthcare practitioners, and policymakers to agree priorities (e.g. rodent damage and potential mitigation); and further research on effects of anthropogenic environmental changes in conjunction with members of communities most likely to be affected by increased contact with wild animals.

## Data Availability

The data on which this research is based are archived on secure servers at the first author's institution. To preserve confidentiality and anonymity of research participants, data are not publicly available.
